# Dr. Ramsbotham's Practical Observations in Midwifery

**Published:** 1821-06-01

**Authors:** 


*Practical Observations in Midwifery; with a Selection of Cases.* Part I. By JOHN RAMSBOTHAM, M. D. Lecturer on Midwifery at the London Hospital, and one of the Physician-Accoucheurs to the Lying-in Charity for delivering poor married Women at their own Habitations. London, 1821; 422 pages, octavo.

NONE of the charitable institutions, with which this metropolis abounds, are more deserving of praise than those established for the relief of parturient women. More than five thousand poor females pass through the "pains and perils of child-birth" annually, under the care of the midwives belonging to the Charity to which Dr. Ramsbotham is attached. This immense number of patients must render difficult and important cases of frequent occurrence, and afford the medical officers the most ample opportunities of observing every variety in the process of parturition. The obstetric art has thus been brought to such a state of maturity, that little improvement can be expected, excepting from those who are engaged in the most extensive practice of it; and we are happy to find that they, whose office it is to assist the birth of mankind, and to relieve some of the miseries, and avert some of the dangers, to which the fairer sex is exposed, are not less zealous in the cause of science than their brethren who have adorned the other branches of the profession by their exertions in our hospitals, fleets, and armies.

The author of the work before us sets out with a brief description of the gravid uterus, the parietes of which he says are neither muscular, tendinous, cartilaginous, nor membranous; but consist of a structure *sui generis* and peculiar to that organ. He next proceeds to describe the process of parturition. He very justly observes that the more gradually and slowly the body and limbs of the child are expelled after the passage of the head, the more perfectly does the uterus contract. This was particularly noticed by Mr. White of Manchester, who advised the expulsion of the child to be retarded in quick labours, to promote and expedite the separation of the placenta.

After the birth of the child it is an excellent rule for the accoucheur to lay his hand upon the abdomen of the patient. He thus avoids the disgrace of leaving her undelivered of a second child in a twin-case; and is enabled to ascertain the degree of contraction the uterus has undergone. The expulsion of the placenta should be left as much as possible to the efforts of nature; but if these should not be sufficient within one hour, it will generally be found that the afterbirth has been detained by some unusual cause, and to require a manual extraction. The occurrence of hæmorrhage might render an earlier and more hasty separation of the placenta requisite; and we know, from experience, that when it has formed a morbid adhesion to the uterine parietes, an occurrence which sometimes happens even in the hourglass-contraction, it is not essential to our ultimate success for us to harrass our patient with attempts to detach all the straggling portions of it which have unavoidably been lacerated, especially when they adhere with firmness, and are of a volume too small to be grasped with the fingers.

The contractile effort of the uterus, which continues after the expulsion of its contents, is generally attended with pain, particularly after the second and subsequent labours. When the pain is distressing an opiate may be given with advantage, in such a dose as merely to moderate its violence; but, when the after-pains continue beyond the second day without fever, we have invariably relieved our patients by giving them an active purge. From this practice Dr. Ramsbotham has also seen the best effects.

A few days after delivery the lactary secretion is established. Dr. Ramsbotham's sentiments on the subject of suckling exactly correspond with those which we have always held.

"A voluntary refusal to suckle on the part of any woman evinces a want of the tenderest feelings, and of maternal affection for her new-born babe. But it does not merely implicate a dereliction of duty; it likewise involves an evasion of the strongest impulses of the human heart: it occasions a transfer of filial affection, gratitude, and obedience from the mother to a hireling, who cannot appreciate their value. Who is prepared to say what may be the future result of this transfer? After a denial of its (his) natural nourishment, after bereaving the babe of its (his) only present birth-right, is it surprising that instances of filial estrangement should occur; or, when once produced, that it should become permanent? May we not attribute some of those disgusting alienations, occasionally met with in certain ranks, to the neglect of this delightful office? Though human institutions admit of ranks and degrees into society, the Divine Will has ordained that all women shall be equally liable to the pains and perils of child-birth, and to its consequences. Milk therefore flows similarly into the breasts of the princess and the peasant, and frequently into those of the former in greater abundance from better fare: it must therefore be repelled or absorbed, under the risk of suppuration and febrile affections, and under the repeated exhibition of nauseous purgatives. That woman but ill consults her future health and comfort, who voluntarily declines this engaging office." P. 72.

After this digression we return to the management of the placenta. Dr. R. has observed an adhesion of it to follow external injuries, which he suspects have not been so considerable as to produce separation, yet sufficient to excite the vessels of the uterus to an undue degree of action, and to throw out coagulable lymph, by which the placental and uterine surfaces are morbidly united. One portion of the placenta sometimes is adherent, while the opposite part is advanced or protruded. In this case care must be taken that neither the mass itself nor the funis be lacerated.

In all instances of adhesion after an uncertain period hæmorrhage ensues, owing to the *partial* separation of the placenta, which prevents the uterine vessels from contracting. A fatal discharge may arise from this source a short time after the birth of the child; and, as a certain quantity of blood must unavoidably be lost by the extraction of the placenta, the sooner this operation is undertaken, perhaps the better. In complete adhesion little or no hæmorrhage is present. The placenta having been withdrawn, it is always desirable that uterine contraction should follow; for while the uterus remains flaccid, it is in danger of concealing an alarming discharge of blood, which distends its cavity, and terminates in death sometimes, before it is suspected. Here a sense of continued faintness is more dangerous than actual syncope: in the former the circulation and hæmorrhage continue; in the latter they are suspended, and coagula are formed at the mouths of the uterine vessels. During a state of absolute syncope, it is the most prudent practice to wait; and, before an attempt is made to remove the placenta, to restore by suitable means the vital energy. The life or death of the woman will, in great measure, depend upon the judgment of the practitioner. Collateral aid may be derived from attention to external circumstances: as cold applications, the admission of cold air, and the administration of acids, together with stimuli for the schneiderian membrane and the stomach, which the attendants will take care to provide, but which ought to be used under the direction of the accoucheur. In hæmorrhage from retained placenta Dr. R. is not an advocate for large and repeated doses of opium. The patient after delivery should not be moved until the faintness has left her. We desire that our patients may remain in the same position for an hour or two: for we consider it better for them to be exposed to the danger of receiving cold from the wet clothes, than of expiring from the syncope, which is liable to follow the least exertion.

Several distressing symptoms usually follow excessive hæmorrhage. The principal of these are a pulsatory pain in the head and a palpitation in the region of the heart and epigastrium. The former subsides in a few days, and Dr. R. says, is relieved by early and active purgatives; the latter we have repeatedly removed by steel, the exhibition of which has been commenced at the end of the second or third week, while dyspnœa and a frequent feeble pulse have been attendant. In these cases, we think, it is probable that venous congestions interrupt the due distribution of the blood, and that the steel unloads the turgid viscera, either by its influence on the quality of the circulating fluids, or by invigorating the muscular structure of the heart and arteries, as in chlorosis inops.

The placenta, after it has been separated from its uterine attachment, may be retained by inactivity of the uterus, or by the hour-glass or the globular contraction. Should any considerable hæmorrhage occur in the first case, it will be requisite to proceed without delay to extract the placenta. It should also be removed in the longitudinal contraction, as soon as the nature of the disease has been discovered; and in the globular one manual assistance will be advisable, when expulsion does not take place within a reasonable time. In the hour-glass contraction we have often found the after-birth adhering to the fundus of the uterus with great firmness; but we believe with Dr. R. that, in general, it is detached from its connexion with that organ, before the morbid contraction happens.

Disruption of the placenta is commonly occasioned by imprudent force applied to the funis; and when a considerable portion of the former is left adhering to the uterus, a corresponding degree of hæmorrhage succeeds. Uterine pain and tenderness commence and terminate in fever, delirium, irregularity of the bowels, offensiveness of the lochial discharge, and sometimes within a week or ten days the melancholy scene is closed by enlargement of the abdomen, restlessness, involuntary evacuations, and death. Sometimes, the symptoms being milder, a puriform discharge has issued daily from the vagina, and been followed by recovery. A gentle attempt should be made to remove the adherent portions of placenta; and should after-pains be troublesome, we must be careful not to interrupt their salutary operation by large doses of opium. Saline medicines and aperients will constitute the principal remedies; and when peritonæal inflammation shews itself, leeches may be safely applied to the abdomen.

It sometimes happens, after the uterus has expelled its contents, and has appeared to have duly contracted, that it suddenly relaxes and acquires from internal hæmorrhage an increase of magnitude, attended with faintness, pallid countenance, frequent pulse, and other marks of depression, which become more and more alarming, until a more permanent contraction occurs, or the woman dies from loss of blood. Corpulent females, of lax fibre, are most liable to this misfortune; also those who have undergone tedious or difficult labours. We remember a melancholy instance of death from this cause, in the practice of a friend, who had just withdrawn into the dining room to take a glass of wine and congratulate the husband on the safe delivery of his wife. On receiving a hasty summons he hurried up stairs, and found his patient, to his great astonishment, in the very act of expiring. Dr. R. throws out some hints respecting the probable cause which deprived the nation of an illustrious and beloved female; but as we are not disposed to wound the feelings of the innocent, we shall abstain from repeating his remarks. Indeed, any detail of suppositious occurrences, which charity ought to bury in oblivion, can neither add to the skill or advantage of the author, nor improve the confidence or good opinion of the public towards the professors of the obstetric art.

The treatment which Dr. R. lays down for relaxation and enlargement of the uterus after delivery, is judicious.

"Apply the hand externally on the uterine tumour, enclose it firmly within the grasp of the hand, and gradually make a firm compression. This practice seldom fails to reduce its size, and to bring on an increased degree of contraction. The external application of cold may also be useful. Ices and cold fluids may be taken at pleasure. Little reliance can be placed on the effects of astringent medidicines, yet they may be properly resorted to. Stimulants, under certain limits, are given with much advantage. If these means fail, the introduction of the hand within the uterus ought not to be deferred ; this is a dernier resort to remove the coagula there accumulated, and to induce uterine action." 195.

"If the introduction of the hand become necessary, it should be retained till uterine contraction be felt; and if the hand be *almost expelled*, the better for the patient." 196.

During this time stimulants become highly necessary.

When collapse comes on after labour, without any apparent cause, the woman says she is extremely ill, the pulse begins to flag, the countenance assumes a pale cadaverous aspect, she becomes restless, and only expresses her feelings by a moan. Soon she is seized with violent pain across the chest, and within an hour or two after delivery expires. No satisfactory account has yet been given of the cause of this occurrence. It appears to Dr. R.

"To consist in a want of accommodation of the several parts within the belly to each other, under the new situation in which they are placed by the abstraction of pressure." 206.

In one of our patients it seemed to be produced by inanition from want of food, in a poor woman, who had a difficult labour occasioned by hydrocephalus internus, which distended the head of the child to a prodigious size.

In a dreadful scene like this, we must not be mere spectators: we must endeavour to restore the vital functions by strong stimulants, as brandy and æther ; and to prevent farther collapse by applying general pressure on the abdomen, and particularly by compression of the uterine tumour.

Protracted labours are divided by Dr. R. into three orders: 1, those which are lingering ; 2, those which cannot be surmounted by the natural efforts ; 3, those which are combined with a relative disproportion between the size of the head and the capacity of the pelvis, through which it is intended to descend.

The first kind of protraction is produced either by an undue degree of resistance in the soft parts, by diminished energy of the propulsive efforts, or an improper position or direction of the head of the child. While the os uteri continues thick, resistent, and incompletely expanded, while the vagina is dry and contracted, and while the external parts shew an indisposition to relax, no assistance from art can be employed with any prospect of success. The exhibition of opium will allay the violent spasmodic pains, which sometimes attend the commencement of parturition, and are not attended with a corresponding effect on the os uteri.

We have, however, frequently found that artificial aid has been required after the use of opium, before the process could be completed. In these cases, however, it is probable that the imperfect and partial energy of the uterus was natural and not induced. When febrile symptoms are evident, accompanied with pain in the head or vertigo, and an unusual rigidity of the os uteri venesection is indicated. The blood should be abstracted from a large orifice in a short space of time, and if possible while the patient is sitting up. The injection of gruel, broth, or other simple warm fluids, into the rectum seems to expedite labour, especially where an accumulation of fæces distends the bowel. The state of the bladder is to be noticed from time to time; and when a tumour, occasioning pain and distress, is found just above the pubes, and distinct from the uterus, immediate relief will follow the introduction of the catheter.

The second genus of protracted labours is the least painful and complicated. Sometimes uterine action entirely ceases, and after an uncertain period returns to complete the process. In our patients, a laxity of fibre and inanimation approaching to mental imbecility have co-existed. Care must be taken to prevent a premature rupture of the membranes, and nature will generally do all that is needful. Dr. R. has had no experience with the ergot of rye, which was brought into notice in this country by Dr. Merriman;* and much doubts the propriety of its frequent administration. We are of opinion that repeated experiments and the most diligent observations will scarcely satisfy an unprejudiced person whether the effects, represented to result from this medicine, do actually proceed from it or from nature. The *post hoc* and *propter hoc*, Dr. R. justly observes, are not always sufficiently distinguished. In these lingering labours the uterus ought to be permitted to expel the whole of the child, that an uniform contraction may be effected, and the placenta detached from its uterine connexion at the same time.

In the third general cause of protracted labour, namely, mal-position of the head, we must be careful not to have recourse to artificial assistance too soon, nor to delay it too long. Twenty-four hours may be allowed to elapse, and after that time the nature of the pains, the state of the woman, and the degree of pressure to which the child has been exposed, must be duly considered. We are by no

---

* See our analysis of Dr. M.'s work on midwifery, in the Med. Chir. Review, for December, 1820.

means advocates for premature assistance; but we have always found in cases of doubt that it has been more advantageous both for the mother and child, when instrumental delivery has been decided upon sufficiently early, than when it has been delayed until the pains have ceased, when it is almost entirely artificial, and consequently more painful to the mother, and more injurious to the infant.

The second order of protracted labours is combined with a slight degree of difficulty. The pelvis may be malformed at the brim, in the cavity, or at the outlet, by diminution of the hollow of the sacrum, by protrusion of the spinous processes of the ischia, by anchylosis of the coccyx, by the approximation of the tuberosities of the ischia, and by want of space in the pubic arch. From whatever cause the difficulty might arise, necessity alone will justify us in the use of instruments. In order to be satisfied that this necessity exists, we must ascertain the state of the os uteri, the degree of the uterine action, the relative size and situation of the head, the length of the time it has remained stationary, the duration of the labour, the pressure on the soft parts, particularly the bladder and urethra, the appearances of the discharges, the degree of permanent pain in the uterus and abdomen, the obvious impression made on the system, the age and constitution of the patient, the state of her mind, the probability of the life or death of the child, and the temperature of the weather, &c. Having determined upon the expediency of employing artificial means, we must endeavour to select the most appropriate instrument. As long as the base of the skull remains above the brim of the pelvis, the short forceps or the vectis cannot be successfully applied, excepting by a few experienced and expert practitioners. In general neither of these instruments can be safely used, until the ear of the child is fairly within the reach of the finger. Dr. R. having been a pupil of the late Dr. Osborne, and having probably accustomed himself to employ the forceps, seems partial to that instrument. We are, however, of opinion, that the vectis will be found capable of effecting every thing which can be accomplished by the short or long forceps; for we have always found it to possess more extractive power than ought ever to be fully exerted, and that it may, when skilfully employed, be as early and successfully introduced as the long forceps can or ever should be. Much disappointment, we apprehend, has arisen to the inexperienced by their observing too implicitly the directions, which are usually given for the application of the vectis. The most advantageous position for this instrument is, we believe, over the angle, the ramus or the symphysis of the lower jaw, according to the position of the head. The occiput is occasionally a good situation; but we have a great objection to the os frontis, on account of the danger of injuring the eyes of the child. Care must always be taken to employ the vectis as an *extractor*, and not as a *lever;* for the latter description of force might occasion such compression on the par vagum and the vessels of the neck of the child, as to produce convulsions or death; and on the urethra of the mother, as may end in sloughing of that part, or the cervix vesicæ. Notwithstanding our partiality for the vectis, we admit that the forceps is the safer instrument in the hands of the young surgeon.

Dr. R.'s directions for the management of those cases in which there is a simultaneous descent of the head and arm, are exceedingly proper.

"When the hand or arm descends into the pelvis by the side of the head, if the accident be discovered in the early part of the labour, before the head has advanced so low as to occupy the cavity, it may be returned without difficulty, by passing two or more fingers of the left hand along the hollow of the sacrum, pushing up the descended part above the brim, and detaining it there till the return of uterine action lowers the head into its place; when this is effected, it rarely descends a second time. In performing this simple operation, no great force is required, if care be taken to give the elbow its natural bend. But if the arm shall happen to come low down in the first instance, and the head be suddenly propelled into the centre of the pelvis pressing upon the arm, this mode of management will seldom succeed; the pressure of the head renders the return impossible. The head must then be allowed to advance under the presence of the retarding arm. When this is the case, the parts of the arm below the point of pressure swell; they suffer all the effects of violent compression from above, which, if the case be long protracted, sometimes produces ulceration and sloughing, after birth. The swelling of the arm is a proof that the child is still alive; yet, by its increasing bulk, it adds to the difficulty." 284.

A portion of pulsating funis descending while the head remains at or above the brim of the pelvis, may sometimes be returned successfully with the left hand of the accoucheur; but Dr. R. most truly says, that it too frequently slides down again.

The most troublesome and dangerous kind of protracted labours are those which are occasioned by a disproportion between the size of the head and the capacity of the pelvis. The most common cause of disproportion will be found in the pelvis itself; and when it does not possess a clear space equal to two inches and three quarters from the symphysis pubis to the prominence of the sacrum, a full-sized head will either not pass at all, or with the greatest difficulty. The operation of *cephalotomia* may thus be required, but should never be performed without a full consultation.

> "The judgment of one individual, however experienced, appears" to Dr. R. "in many instances, hardly sufficient to authorise the operation. Perforation of the infantile head is, to say the least of it, a horrible proceeding, from which every man would be glad to refrain if he possibly durst; and which he would always wish to defer, as long as a sense of professional duty, and the safety of the mother, allowed." 310.

When the malformation is known or easily detected, the perforation is to be made as early during the labour as the state of the os uteri and of the soft parts will permit the safe application of the perforator. The contents of the cranium being evacuated, the extraction may be deferred for some hours, to allow of greater collapse and accommodation. When, however, the pelvis is much deformed, and the pains become strong soon after perforation, it is the best practice to proceed to immediate extraction, in order to avail ourselves of the powerful aid of the expulsive efforts. Dr. R. has commonly used the crotchet, and says that in those cases in which he has tried Dr. Davis's craniotomy-forceps, he has been much pleased with their extractile effects.

The practice of inducing premature labour will, in most cases, excepting when the deformity is extraordinary, have the effect of saving the life of the child, or at least of mitigating the sufferings of the mother. Dr. R. very properly ridicules the vulgar prejudice respecting the improbability of an eight months' fœtus being likely to live.

The uterus from the violence of its own contractions is liable to tear itself asunder. This dreadful and commonly fatal accident happens suddenly and without previous warning. In a preternatural presentation, the child with or without the placenta may be expelled into the abdominal cavity by the action of the fundus uteri. A rupture of the peritonæal coat of the uterus sometimes occurs without affecting the uterine structure. All the symptoms of actual rupture of the uterus, excepting those connected with the escape of the child, are observable in a diminished degree. A breach of the vagina also occasionally takes place, and, when extensive, is followed by symptoms much resembling those arising from uterine rupture.

Rupture of the bladder arises from inattention, is attended with many of the symptoms attendant on lacerated uterus, and is equally fatal.

Dr. R. suspects that rupture of the uterus is preceded by some injury that organ has sustained in a previous labour, and we are decidedly of the same opinion.

"I have never met with a rupture of the uteurs in a first lying-in. The accident has happened, in those cases which I have seen, in a subsequent labour, and sometimes after several difficult births, though living children have been expelled. I am thence led to suspect, either that the uterus has received some local mechanical injury from the violence of its own efforts, or from the previous effects of artificial assistance, by which its structure is, at this point, weakened; or, that it is thinned at the part where it gives way during the last months of gestation, by continued pressure against some prominent part of the pelvis.

"The breach of structure usually happens somewhere about the cervix; either anteriorly towards the symphysis pubis, or posteriorly towards the prominence of the sacrum. The rent is either transverse, or is carried laterally upward. The fundus uteri rarely gives way; yet its body and sides occasionally do." 383.

This accident may arise from an injudicious or violent endeavour to overcome the uterine contraction in a preternatural presentation; or it may spontaneously occur before labour is perfectly established, or as early as the fifth month of pregnancy.

Every case of ruptured uterus which Dr. R. has seen, has sooner or later proved fatal. Some women barely survive delivery; some live several days after it; and a few cases are on record, in which recovery has followed. It is proper, in every instance, to afford the mother a chance of restoration by as early a delivery as may be practicable. The placenta should also be speedily removed; and when it is necessary to introduce the hand for that purpose, the rent in the uterus may often be discovered by the fingers. An opiate, and afterwards such medicines as the symptoms may require, should he administered. The immediate distress having subsided, inflammatory action supervenes, and at an uncertain period a collapse of the physical powers comes on, which terminates existence. This being almost invariably the consequence of rupture, Dr. R. thinks it may be a question worthy the consideration of the profession, whether the cæsarian section ought not occasionally to be substituted for the perforation of the head.

We cannot do justice to the work before us without taking some notice of the valuable and well selected cases, which illustrate the different subjects, and occupy a very considerable portion of the volume. The younger members of the profession will find them replete with practical information and many useful hints; and those who have passed a long life in the practice of midwifery, and suppose themselves to be competent to manage the most desperate cases, will derive infinite satisfaction, *if they can be invited to read cases*, from observing the course which has been pursued by Dr. R. in situations which do not frequently present themselves in the most extensive private practice. The colloquial style which Dr. R. has adopted in relating his cases, has led him, no doubt unintentionally, into a tedious detail of uninteresting minutiæ, which swell the book, increase its price, and detract from its value. For instance, of what advantage is it to us to be told whether a chaise or a hackney-coach was sent to the door, or the doctor was obliged to travel on foot; whether the husband or man-midwife happened to fetch him in a hurry, or he received a note from a pothering old woman? We have another complaint to urge against Dr. R. which is, that he is occasionally too severe with his obstetric brethren. He should not rely too much on his advantages; nor, from being too often consulted by midwives and ignorant and timid practitioners, be induced to entertain a contemptible opinion of the rest of the profession. There is hardly a country-town in the kingdom, containing half a dozen surgeons, in which there is not, at least, one capable of deciding and acting in cases of emergency, both skilfully and successfully. These remarks originate in pure friendship to the author, whose work we have perused with great satisfaction and improvement, and can recommend as a safe and sure guide to successful practice. The unfortunate occurrences, as well as the favourable ones, are brought to view with extreme candour; and the dangers and difficulties are in all cases diminished by the excellent and decisive rules which are laid down. We are indeed so much pleased with Dr. R.'s practice, that we shall be quite impatient for the appearance of the second part of his work; and if he should receive our observations as kindly as they are intended, we think he will see the advantage of compressing into the body of his next book all the useful and valuable information he possesses, with the view of avoiding the introduction of cases, which are seldom read, excepting they are few, important, and condensed. The multiplication of books on every subject should lead authors to keep in view the old adage: μεγα βιβλιον μεγα κακον, "a great book is a great evil."

We have thus presented our readers with a view of Dr. Ramsbotham's book, which we hope will induce them to read it with attention, not neglecting to peruse the cases, which we must repeat contain most valuable facts; and we take leave of the author for the present in perfect good humour, hoping it will not be long before we shall receive as much pleasure in displaying the beauties of his next production, as we have felt pain in exposing the few and trifling imperfections in the one before us.

> " Verùm ubi plura nitent in carmine, non ego paucis
> " Offendor maculis, quas aut incuria fudit,
> " Aut humana parum cavit natura."
>
> *Q. Hor. Flacc.*

